# Response of adult *Cochliomyia macellaria*, *Musca domestica*, and *Sarcophaga bullata* (Diptera: Calliphoridae, Muscidae, Sarcophagidae) to odors produced by commercial fly baits in a two-choice olfactometer bioassay

**DOI:** 10.1093/jisesa/ieaf020

**Published:** 2025-04-08

**Authors:** Ann L Carr, Steven S Denning, Anastasia C Figurskey, Kim Y Hung, Michael H Reiskind, David Wes Watson

**Affiliations:** PathGroup, Nashville, TN, USA; Department of Entomology & Plant Pathology, Box 7613, North Carolina State University, Raleigh, NC, USA; Global RD&E Pest Control, SC Johnson Center for Insect Science and Family Health^TM^, Wind Point, WI, USA; Coachella Valley Mosquito & Vector Control District, Indio, CA, USA; Department of Entomology & Plant Pathology, Box 7613, North Carolina State University, Raleigh, NC, USA; Department of Entomology & Plant Pathology, Box 7613, North Carolina State University, Raleigh, NC, USA

**Keywords:** Ecology & behavior, chemical ecology, veterinary entomology

## Abstract

We developed a two-choice spatial olfactometer to evaluate the response of adult secondary screwworm (*Cochliomyia macellaria*), house fly (*Musca domestica*), and flesh fly (*Sarcophaga bullata*) to two commercially available fly-trap attractants, Captivator and FliesBeGone in three-dimensional space. Liquid fly baits were prepared according to the manufacturer’s recommendations and aged to discern the relative attraction of fresh and older baits. Each 0.07 m^3^ (2.5 ft^3^) arena was fitted with two fresh air intake ports, collection chambers containing the attractant or a blank control, and air exhaust ports. We released adult flies into an arena with sufficient space to allow free flight and response to the test attractants. Each comparison was replicated eight times with fresh flies. Flies were more responsive to commercial bait than the water control. Air flowrates, as measured through the intake ports, was determined to be a limiting factor for *C. macellaria* and *S. bullata* with significant responses rates observed to flowrates ≤0.14 m^3^/min (5 ft^3^/min) and ≤0.25 m^3^/min (9 ft^3^/min), respectively. In contrast, *M. domestica* appeared to respond similarly to all flowrates tested (≤0.31 m^3^ (11 ft^3^/min). In direct comparisons with a water control, *M. domestica* was attracted to baits regardless of bait age. In similar experiments, *C. macellaria* was significantly responsive to FliesBeGone aged 2 and 3 d but not Captivator regardless of age. Lastly, *S. bullata* was most responsive to FliesBeGone aged 3 and 4 d, and Captivator aged 4 d. Female flies responded to fly baits more frequently than males.

## Introduction

Filth flies, including blow flies (Diptera: Calliphoridae), house flies (Diptera: Muscidae), and flesh flies (Diptera: Sarcophagidae), use a variety of solid wastes including discarded food, excrement and carrion, for nutrients and oviposition (Olsen 1998). The association of filth flies with unsanitary and potentially contaminated nutrient sources and breeding locations facilitates the mechanical transmission of disease agents to susceptible humans and animals. Nuisance behaviors common to filth flies, such as entering houses and visiting prepared foods, amplify their health impacts as pathogen vectors. Filth flies are also problematic for largescale livestock and poultry farms where high fly populations can significantly affect overall animal wellness (Gerry et al. 2011) and pose a food biosecurity threat resulting in legally mandated fly control (Thomas and Skoda 1993, Royden et al. 2016). Integrated pest management strategies to reduce filth fly populations in part utilize insecticidal surface sprays, toxic baits, and nontoxic baited traps. With increasing filth fly resistance to commonly used neonicotinoid and pyrethroid insecticidal sprays, reliance on effective toxic baits and nontoxic baited traps has increased (Scott et al. 2013, Heath and Levot 2015). The integral component of both toxic baits and nontoxic baited traps for filth fly management is a robust and odorous attractive bait. Attractive baits exploit the natural dependence of filth flies on volatile organic compounds (VOCs) to locate nutrients and breeding locations within the proximate environment.

Laboratory bioassays are a fast and inexpensive approach for screening odorous compounds and evaluating the corresponding fly behaviors. However, most laboratory bioassays involve the simultaneous testing of visual and olfactory cues and provide no means of distinguishing filth fly attraction by means of vision and/or olfaction (Chaudhury et al. 2015, Upakut et al. 2017). Data from such bioassay methodologies make it difficult to distinguish between compounds with purely olfactory activity that do not require synergistic visual cues for attraction. It may also lead to a sex-biased response that may not be representative of the odorous attractant’s chemical ecology. Furthermore, few bioassay tools accommodate more than one filth fly species while only requiring minor system and protocol adjustments. Recently, Flint and Tomberlin (2021) developed a dual-choice olfactometer to measure responses of secondary screwworm flies to glass jars provisioned with and without beef liver. Expanding upon this concept, we developed an efficient laboratory bioassay for assessing filth fly olfactory behavior in response to attractants, allowing us to conduct many comparisons across multiple species with sufficient replication to make strong inferences of relative attractiveness.

We evaluated the response of three filth fly species, *Cochliomyia macellaria*, *Musca domestica*, and *Sarcophaga bullata* in a two-choice laboratory olfactometer to high performance commercial fly baits. The two commercial fly baits, Captivator and FliesBeGone, were selected for evaluation based on their reported capacity to attract filth flies outdoors (Diclaro et al. 2012, https://www.starbarproducts.com/all-products/traps/captivator-fly-trap, and https://fliesbegone.com/). Olfactometer protocols were standardized for each of the three filth fly species and species-specific attractant behavior to the commercial fly baits successfully documented.

## Materials and Methods

### Flies

Adult *C. macellaria*, *M. domestica*, and *S. bullata* were obtained from locally established laboratory colonies. Flies were housed in an insectary maintained at 26 °C and 70% relative humidity with a photoperiod of 16-h light: 8-h dark, with dusk and dawn periods of 1 h each at the beginning and ending of the scotophase. Flies were provisioned with water, granulated sucrose, and powdered milk ad libitum. All flies used for olfactometer bioassays were 4-14 d postemergence and maintained in cages containing mixed sex colonies with continued access to water and granulated sucrose.

### Commercial Baits for Olfactometer Bioassays

We screened the commercial fly baits Captivator (Starbar, Schaumburg, IL) and FliesBeGone (FliesBeGone, Tom River, NJ) in olfactometer parameter optimization bioassays and experimental bioassays. To evaluate the longevity of each commercial fly bait, and their respective attractiveness to *C. macellaria*, *M. domestica*, and *S. bullata*, each bait was prepared according to manufacturer’s instructions in separate ventilated containers and placed into a dark incubator at 30 °C for a period of 7 d. Aliquots from each commercial bait were extracted into labeled sterile 50 ml conical tubes at 0, 1, 2, 3, 4, and 7 d, and stored at −20 °C to suspend the aging process or bacterial growth until used in olfactometer bioassays. In total 12 aliquot samples were collected, 6 samples from Captivator and 6 samples from FliesBeGone, and referred to as such: 0 d (d = day old) Captivator, 1 d Captivator, 2 d Captivator, 3 d Captivator, 4 d Captivator, 7 d Captivator, 0 d FliesBeGone, 1 d FliesBeGone, 2 d FliesBeGone, 3 d FliesBeGone, 4 d FliesBeGone, 7 d FliesBeGone. All commercial baits used were purchased 2 wk preceding laboratory olfactometer bioassays and stored accordingly in a cool dry location.

### Laboratory Airflow Rate Standardization and Bioassay Optimization

Preliminary behavioral bioassays were conducted with the respective commercial fly baits (Captivator and FliesBeGone) to optimize olfactometer protocols and standardize air flowrates for *C. macellaria*, *M. domestica*, and *S. bullata*, respectively. Tests were conducted during the daytime between the hours of 11 AM and 4 PM at 24 ± 2 °C with a relative humidity of 40% under ambient (fluorescent) lighting. Ambient lighting was sufficient to elicit robust behavioral responses from *C. macellaria* and *M. domestica* flies. In preliminary trials, we noted *S. bullata* required supplemental olfactometer arena lighting, so an individual 13 W halogen light was used to illuminate each individual olfactometer arena during bioassays with *S. bullata*.

**Fig. 1.**
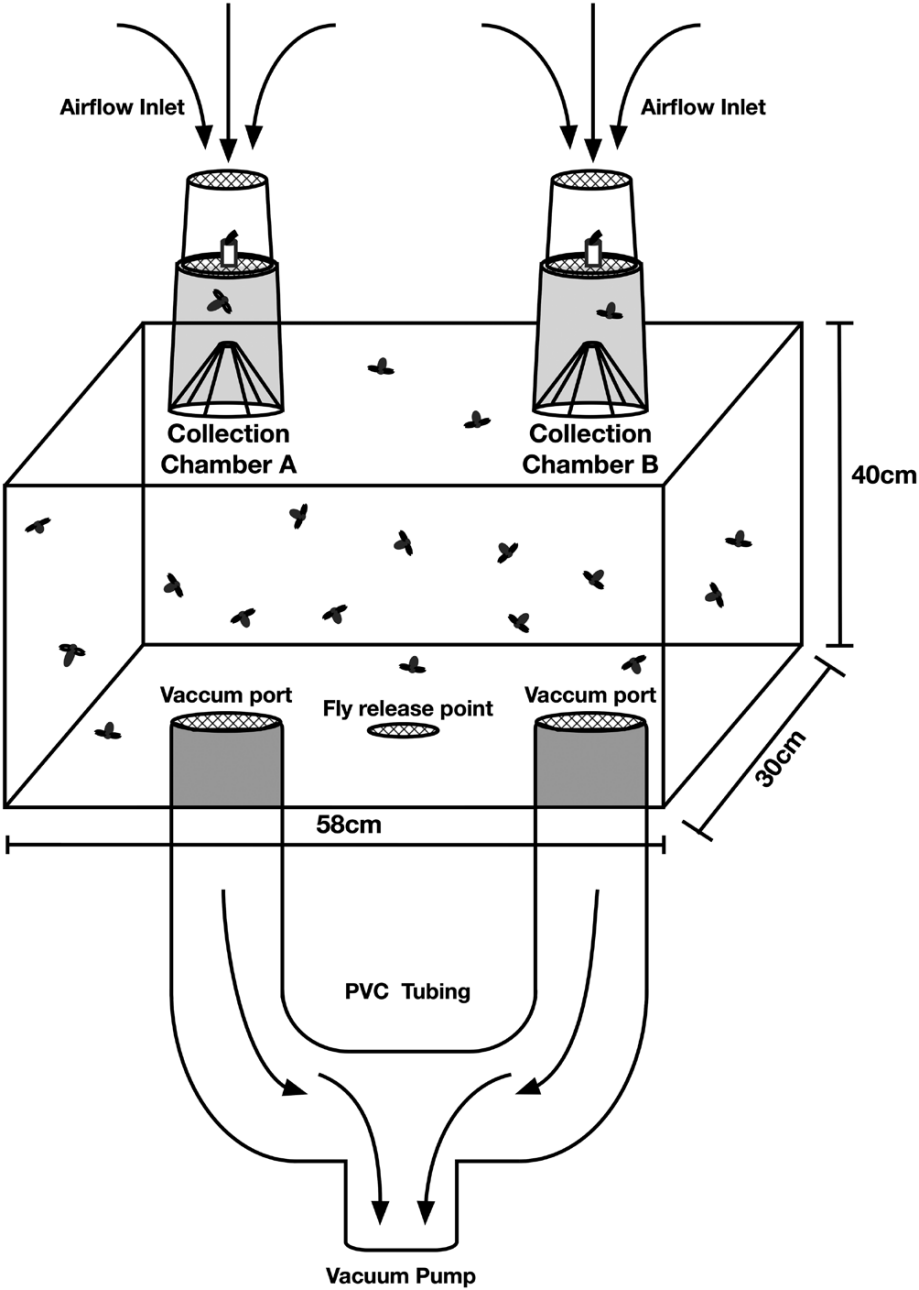
Diagram of olfactometer apparatus used in laboratory bioassays of commercial fly baits. Airflow within the arena was generated by means of a vacuum pump controlled by a variable autotransformer that pulled outside air in through the inlet ports, across the vials containing test materials, into the arena containing the test subjects and subsequently exhausted out of the arena through vacuum port attachments in the base. Air flowrate was measured using an anemometer inserted into the exhaust piping downstream from each arena.

**Fig. 2.**
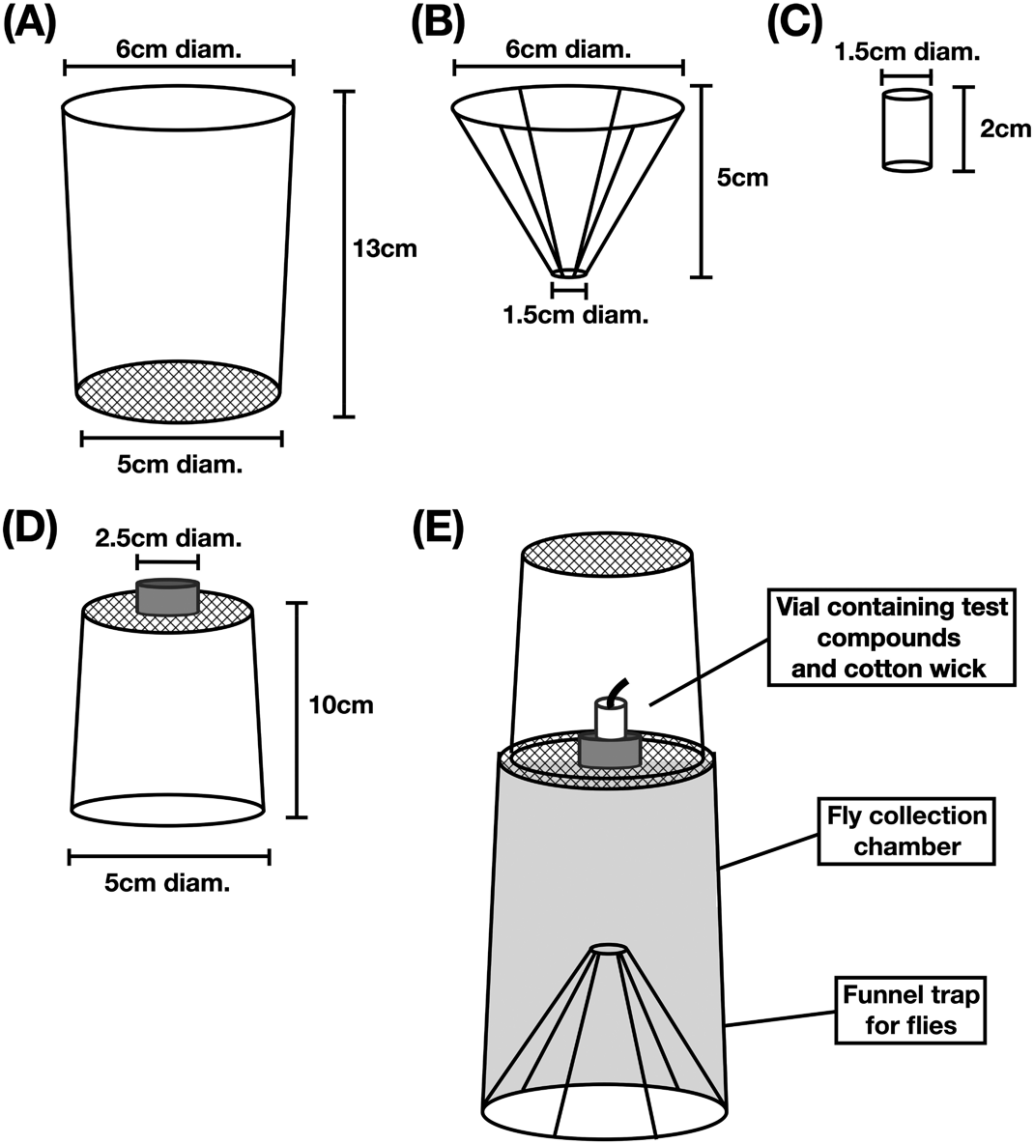
Diagram of olfactometer apparatus “collection chamber” and assembly parts used in laboratory bioassays of commercial fly baits: A) ventilated collection jar; B) funnel that acted as a trapping device; C) single test tube holder; D) ventilated cylinder for directing airflow and fixed tube holder; and E) assembled “collection chamber”.

Olfactometers were constructed as pull, two-choice bioassay systems (Fig. 1). Eight individual olfactometers were constructed for bioassay experiments to generate data representing distinct biological replicates. Four replicate olfactometers were used for each session, once in the morning and replaced with 4 clean olfactometers for the afternoon experiments. Each olfactometer consisted of a rectangular arena (30 cm W × 40 cm H × 58 cm L) with a removable base. The air volume (space) within each olfactometer arena was 0.07 m^3^. Two vacuum ports and a sealable central inlet for fly release were located on the base of each olfactometer arena (Figs. 1 and 2). The base of the arena was sealed with a gasket and clamps to prevent air leaks and create an airtight environment. Two removable collection chambers served as the air inlet ports and point of odorant release, were affixed to the top of the olfactometer arena (Figs. 1 and 2). Collection chambers comprised a 13 cm tall container with a 5 cm tall funnel inserted into the mouth of the container to act as a trapping device for flies entering into the set container (Fig. 2A, B, and E). Additionally, the bottom of the collection chamber was fitted with a metal mesh (window screen) to permit airflow (Fig. 2A and E). A 1.5 ml microfuge vial (Thermo Fisher Scientific, Nashville, TN, USA) containing a cotton wick placed into the tube holder affixed to the upper surface of the collection chamber’s metal screen acted as the delivery mechanism for odorants during olfactometer bioassays (Fig. 2D and E). To maximize the efficiency of the odorant delivery mechanism, airflow was directed over the vial wick by means of a removable 10 cm tall cylinder, with a metal mesh top, that attached to the “collection chamber” fitting over the vial stand (Fig. 2D and E). A vacuum system (eg, oil-free vacuum pump or an industrial shop vacuum) controlled by a variable autotransformer (ISE, Cleveland, OH), attached to the arena vacuum ports by means of PVC (poly vinyl chloride) piping in a “T” configuration downstream from the arena, was used to pull air into the arena via the inlet ports generating airflow for olfactometer bioassays (Fig. 1). Vacuum pressure controlled by the variable autotransformer functioned as a flowrate controller. A hot-wire anemometer (Testo, West Chester, PA) was used to carefully monitor and record all airflow rates was inserted through a sealable port to measure flowrates as the air exited the arena. Given the construction of an airtight bioassay arena, we assume that the airflow rate entering into a single olfactometer inlets port is equal to half the airflow rate documented as exiting the olfactometer vacuum port. Fresh outside air entered the system, passed over the test attractant, through the collection chamber, entering the fly release arena and out through ports in the bottom. Air and any associated odors removed from olfactometer was exhausted out of the testing area.

In preparation for protocol optimization and flowrate standardization bioassays, each of the 8 collection chambers, 2 per olfactometer, were fitted with a sterile funnel and attached to the olfactometer with gloved hands. Olfactometer bioassays were conducted as two-choice assays, with one treatment chamber and one control chamber. For preliminary experiments, treatments were either 4 d Captivator or 3 d FliesBeGone paired with distilled water controls. A standard volume (1 ml) of either 4 d Captivator or 3 d FliesBeGone, according to the test fly species, was pipetted into 4 1.5-ml vial (microfuge tubes) containing cotton wicks. The same procedure was used to prepare 4 1.5-ml vials with cotton wicks containing distilled water to determine if flies responded equally to chambers A and B with a water only treatment, which demonstrated no bias between chambers in any of the 8 olfactometers (Fig. 1).

All test vials (1.5 ml microfuge tubes), used in olfactometer bioassays were tightly sealed and weighed prebioassays and postbioassays to obtain precise records of release rates. Test vials were only unsealed during olfactometer bioassays to prevent room contamination of odorants and potential loss of solution that could impact release rate calculations. Each olfactometer had one treatment test vial placed into the vial stand of collection chamber A and one control vial placed into the vial stand of collection chamber B (Fig. 1). Controls were always prepared first to prevent cross contamination of chemicals during handling and bioassay set-up. The location of treatments and controls were switched between the two collection chamber positions (A and B) to avoid positional response bias in each subsequent assay (Fig. 1). All parts were disassembled and washed daily with hot water and Micro-90 detergent (International Products Corp., Burlington, NJ) followed by a 95% ethanol rinse. To determine if all 3 species responded similarly to multiple air flowrates, (0.08, 0.14, 0.20, 0.25, and 0.31 m^3^/min (3, 5, 7, 9, and 11 ft^3^/min)), we evaluated each species separately. *C. macellaria* was tested against 0.08, 0.14, and 0.20 m^3^/min, *M. domestica* 0.08, 0.14, 0.20, 0.25, and 0.31 m^3^/min, and *S. bullata* against 0.08, 0.14, 0.20, and 0.25 m^3^/min. All described airflow rates represent the rate of air exiting the arena via the vacuum port. All described airflow rates represent the rate of air exiting the arena via the vacuum port. We assumed that the airflow rate through a single inlet port is equal to half the airflow rate exiting the arena through the vacuum port, but did not measure each inlet port. Following airflow optimization experiments, bioassays were performed utilizing only distilled water treatments and the flowrates 0.08 m^3^/min (0.04 m^3^/min/port) for *C. macellaria* and *S. bullata* trials and 0.19 m^3^/min (0.09 m^3^/min/port) for *M. domestica* experiments to determine if flies responded equally to chambers A and B with a water only treatment which demonstrated no bias between chambers in any of the 8 olfactometers (Fig. 1).

Each replicate for each bait comparison was a cohort of flies (*n* = 30 of *C. macellaria* and *S. bullata*, 50 for *M. domestica*) in a single olfactometer. Each comparison had 8 replicates. Flies were acclimated to experimental conditions for a minimum of 30 min prior to being transferred into olfactometers. After placement of the treatment, control vials, flies of one species were released into the olfactometer via the central base inlet. Bioassays testing *C. macellaria* adults used 30 randomly selected flies with an equal male:female ratio. Bioassays testing *M. domestica* adults used 50 randomly selected flies also with an equal male:female ratio. Carbon dioxide gas (AirGas, Morrisville, NC) was used to anesthetize *C. macellaria* and *M. domestica* for sexing and counting. The carbon dioxide gas had limited effects on *M. domestica* adults, and flies were permitted a recovery period of 30-60 min prior to transportation into the bioassay test environment for acclimation. *C. macellaria* adults were susceptible to the side effects of the carbon dioxide gas and required a minimum 4 h recovery period prior to transportation into the bioassay test environment. *S. bullata* proved the most difficult to prepare for olfactometer bioassays. Carbon dioxide gas had injurious effects on *S. bullata* adults and so refrigerated chilling was used to count and sex *S. bullata*. Additionally, *S. bullata* test subjects that were randomly selected from cage colonies performed very poorly in olfactometer experiments. To improve bioassay response rates, test vials were replaced with raw liver to pre-select *S. bullata* that were actively searching for nutrients or oviposition sites; care was taken to prevent *S. bullata* from directly contacting the liver (Fig. 1). Bioassays testing *S. bullata* adults used 30 randomly selected flies from the batch of liver preselected flies with an equal male:female ratio. Bioassays were conducted for 30 min when testing *C. macellaria* or *M. domestica*, and 45 min for *S. bullata* and replicated a minimum of 8 times with flies not previously tested.

### Laboratory Bioassays of Commercial Baits

Experimental bioassays of commercial baits were conducted using the olfactometer system and methods described above. Tests were conducted during the daytime between the hours of 11 AM and 3 PM at 24 ± 2 °C with a relative humidity of 40% under ambient (fluorescent) lighting. Supplemental lighting (13 W halogen bulbs) was added to illuminate olfactometer bioassays while testing *S. bullata* flies. Samples of the aged commercial fly baits Captivator and FliesBeGone were tested against *C. macellaria*, *M. domestica*, and *S. bullata* in olfactometer bioassays. Airflow, based on the results of flowrate standardization experiments was set to 0.08 m^3^/min (0.04 m^3^/min/port) for *C. macellaria* and *S. bullata* trials and 0.19 m^3^/min (0.09 m^3^/min/port) for *M. domestica* experiments. The entire air volume of the olfactometer, 0.07 m^3^, was exhausted in totality every 50 s for bioassays with *C. macellaria* and *S. bullata*, and every 21 s for bioassays with *M. domesti*ca. Olfactometer bioassays were conducted as two-choice assays, with one treatment chamber (commercial bait) and one control chamber (distilled water). Treatments and controls were rotated between the collection chamber positions (A and B) to prevent positional response bias. Additionally, experiments were conducted utilizing only controls in the collection chamber positions to eliminate the possibility of false positive responders to physical aspects of the olfactometer system. All equipment was disassembled and washed between assays. All flies captured in collection chambers were recorded as positive responders and attracted to test chemical or the control. Flies that remained in the olfactometer were considered non-responders. Final release rates for all tested aged samples of Captivator and FliesBeGone were 2 ± 0.2 and 1.5 ± 0.3 mg/min, respectively.

### Statistical Analysis

All flies that were captured in collection chambers were recorded as positive responders and attracted to either treatment or control solutions. Response data from bioassay experiments was the count of flies by sex (so out of 15 for *S. bullata* and *C. macellaria*, 25 for *M. domestica*) responding within each of the 8 replicates and thus we modeled the behavioral response with a Poisson regression. We tested whether *Treatment*, *Day*, *Cage Location (1*, *2*, *3*, *or 4), and Chamber Position (A or B)* affected fly response*. Day*, *Cage Location*, *and Chamber Positions* were blocking factors for the Latin Square experimental design used to investigate *Treatment* effect on the number of flies attracted to each commercial bait (Supplemental Material Table S1). Response data from bioassay experiments were converted to percentages of the total number of flies making a choice of commercial baits and water controls. Percentages were compared in a analysis with an expected probability for chamber choice of 0.5 for both test and control materials. Count data of male and female responses to commercial baits and water controls were compared using a generalized linear model with Poisson regression (* *P* < 0.05). All analyses used the statistical functions package of the python SciPy API (Virtanen et al. 2020).

## Results

### Flowrates and Controls

**Fig. 3.**
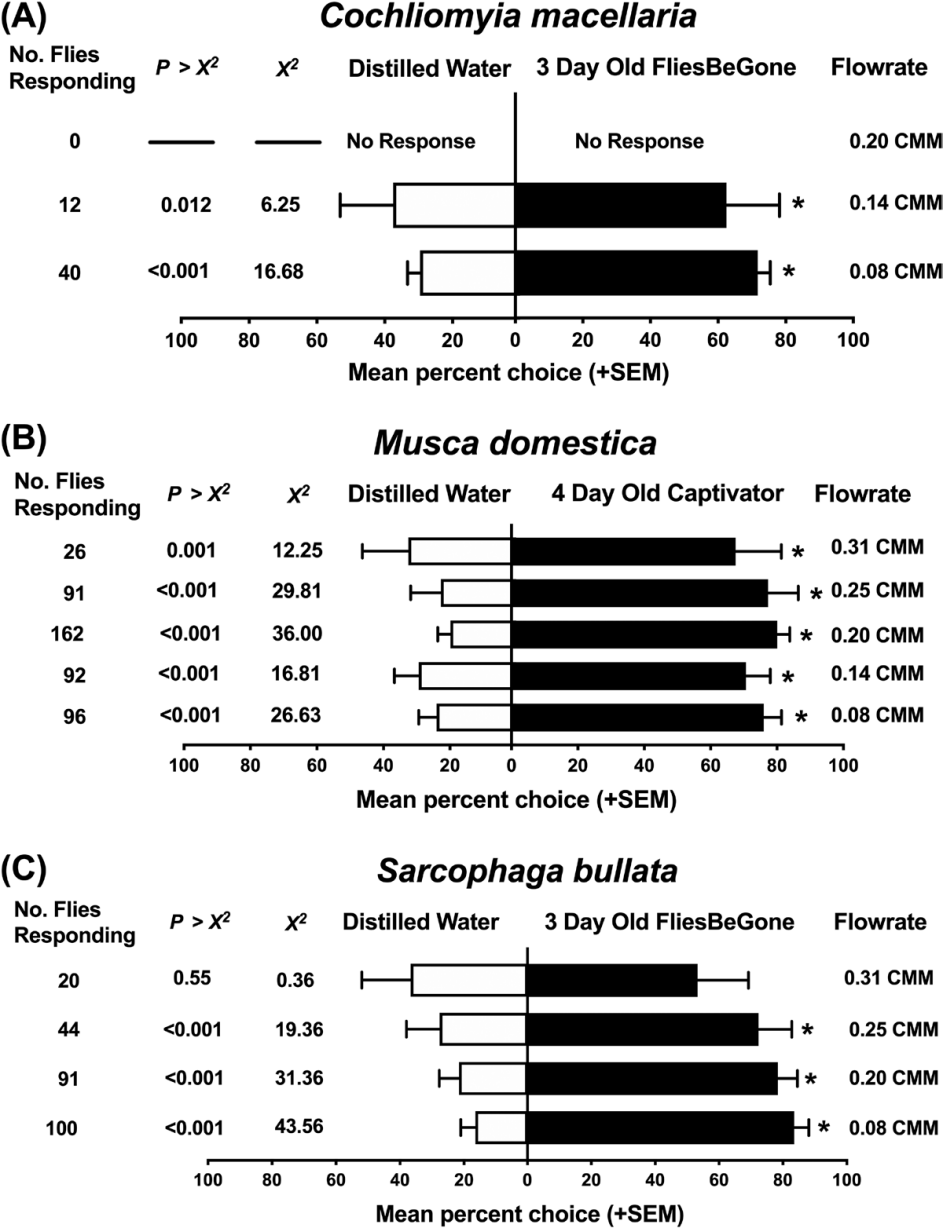
A) Mean response (+SEM) of *Cochliomyia macellaria*, B) *Musca domestica*, and C) *Sarcophaga bullata* flies in laboratory olfactometer airflow standardization bioassays testing either 4 d Captivator or 3 d FliesBeGone with a distilled water control and varying airflow rates recorded in m^3^/min (CMM). The observed number of responding flies (no. of flies responding) represents the combined count of 8 distinct biological replicates for each experimental flowrate. Statistical analyses were performed using *χ*^2^ analysis and Poisson regression (*P < 0.05).

**Fig. 4.**
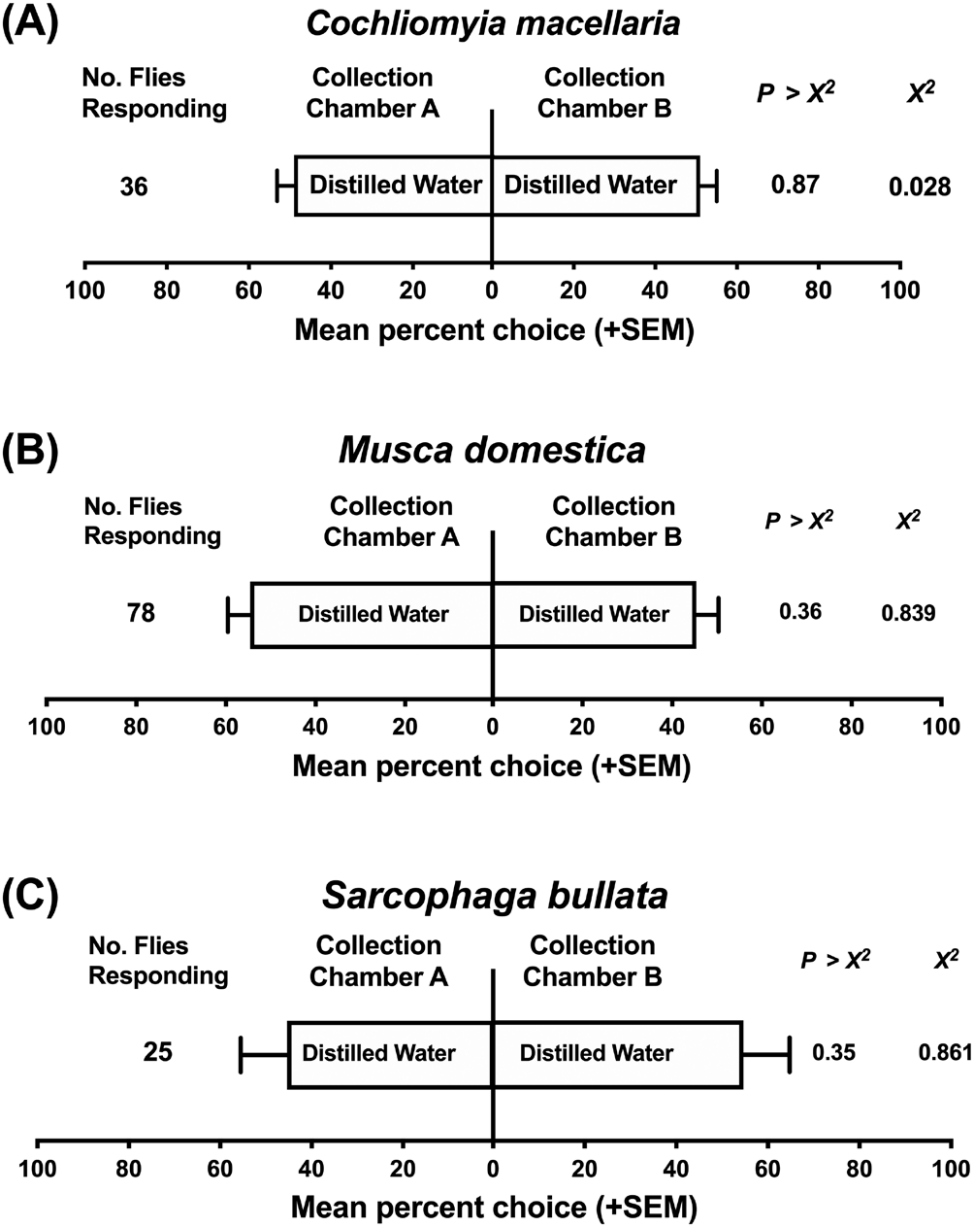
A) Mean response (+ SEM) of *Cochliomyia macellaria*, B) *Musca domestica*, and C) *Sarcophaga bullata* flies in laboratory olfactometer bioassays testing distilled water controls. Flowrates used were 0.08 m^3^/min for *C. macellaria* and *S. bullata* trials and 0.19 m^3^/min for *M. domestica* experiments. The observed number of responding flies (no. of flies responding) represents the combined count of 8 distinct biological replicated for each distilled water treatment. Statistical analyses were performed using *χ*^2^ analysis (*P < 0.05).

Airflow rates significantly affected the response of *C. macellaria*, *M. domestica*, and *S. bullata* in olfactometer bioassays (Fig. 3). For *C. macellaria* and *S. bullata*, higher flowrates greatly reduced the total number of flies responding and the overall preference for the commercial baits compared to the distilled water control. In olfactometer bioassays, *C. macellaria* and *S. bullata* were most responsive to flowrates ≤0.14 and ≤0.25 m^3^/min, respectively, while *M. domestica* responded well to all flowrates (Fig. 3). *C. macellaria* flies responded significantly to commercial baits at the lower flowrates 0.08 m^3^/min (*χ*^2^ = 16.68, df = 1, *P* < 0.0001) and 0.14 m^3^/min (*χ*^2^ = 6.25, df = 1, *P* = 0.012), compared to the moderate flowrate 0.20 m^3^/min which elicited zero responding flies. *S. bullata* responded significantly to commercial baits at the lower and moderate flowrates: 0.08 m^3^/min (*χ*^2^ = 43.56, df = 1, *P* < 0.0001), 0.20 m^3^/min (*χ*^2^ = 31.36, df = 1, *P *< 0.0001), and 0.25 m^3^/min (*χ*^2^ = 19.36, df = 1, *P* < 0.0001), with responses, both preference and total number of responding flies, diminishing at the higher flow 0.31 m^3^/min. *M. domestica* attraction to 4 d Captivator was significantly greater than distilled water regardless of the flowrate, though flies were slightly more responsive to moderate airflow of 0.20 m^3^/min (Fig. 3). *C. macellaria* and *S. bullata* flies exhibited the highest total number of responding flies with significant attraction to 3 d FliesBeGone during bioassays testing the lowest flowrate (0.08 m^3^/min), respectively (*χ*^2^ = 16.68, df = 1, *P *< 0.0001; *χ*^2^ = 43.56, df = 1, *P *< 0.0001). Experiments evaluating distilled water controls in both ports in the olfactometer bioassay system identified no biases in preferences (Fig. 4).

### Commercial Baits

**Fig. 5.**
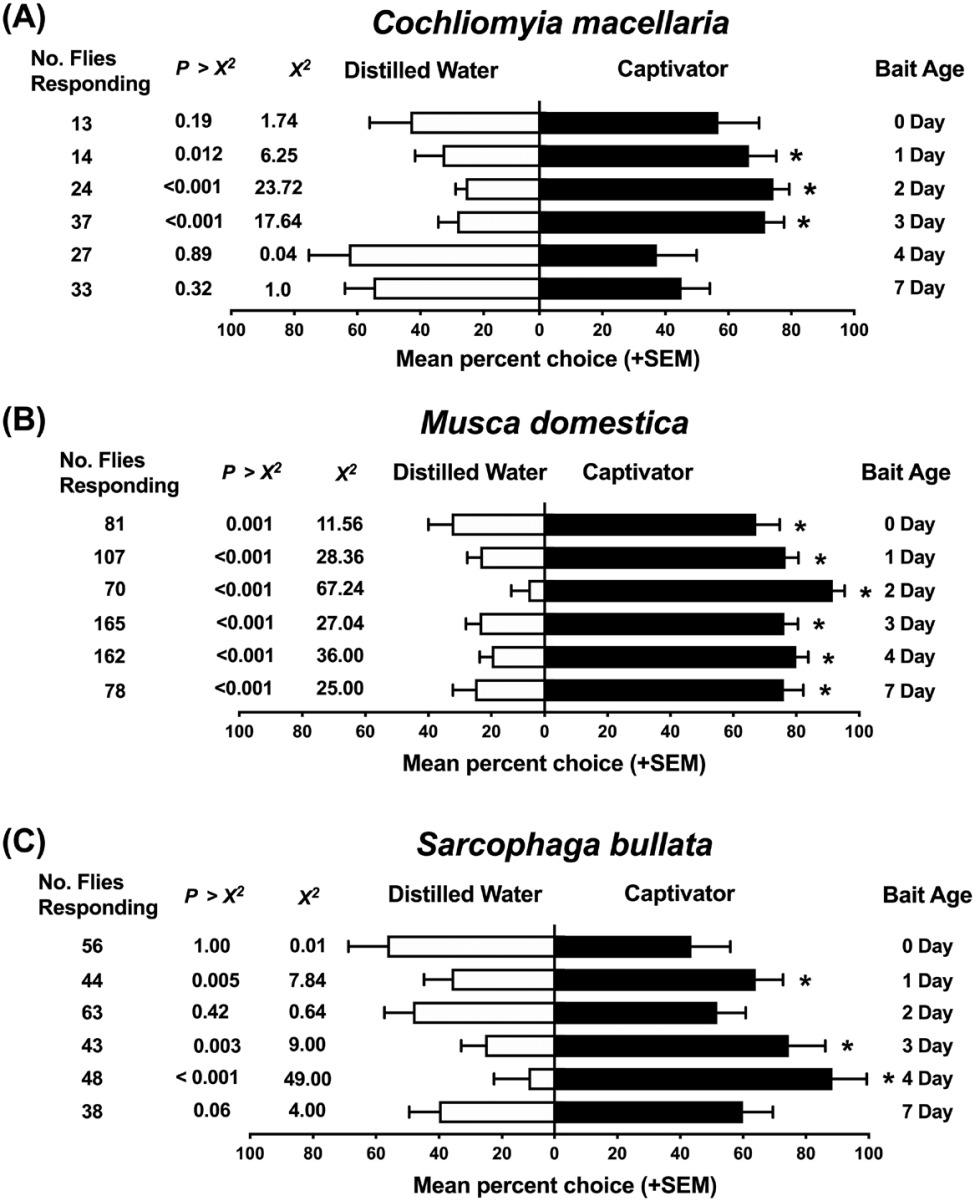
A) Mean response (+SEM) of *Cochliomyia macellaria*, B) *Musca domestica*, and C) *Sarcophaga bullata* flies in laboratory olfactometer bioassays testing 6 distinct ages of the commercial fly bait Captivator, prepared according to the manufacturer’s protocol, with a distilled water control. Flowrates used were 0.08 m^3^/min for *C. macellaria* and *S. bullata* trials and 0.19 m^3^/min for *M. domestica* experiments. The release rate of Captivator was 2 ± 0.2 mg/min for all ages tested. The observed number of responding flies (no. of flies responding) represents the combined count of 8 distinct biological replicated for each distilled water treatment. Statistical analyses were performed using *χ*^2^ analysis (*P < 0.05).

*C. macellaria* showed no significant preference for 1, 2, and 3 d Captivator relative to distilled water in the olfactometer bioassays (Fig. 5A). Female *C. macellaria* responses were significantly greater than males to Captivator baits aged 2, 3, and 7 d (Table 1). *S. bullata* responded significantly to Captivator bait aged 4 and 7 d (Fig 5C) and females significantly responded to baits aged 2, 3, and 4 d (Table 1). *M. domestica* were significantly attracted to all ages of Captivator in olfactometer bioassays (Fig. 5B), with 4 d Captivator compared to distilled water having the highest response rate (*χ*^2^ = 36.00, df = 1, *P *< 0.0001) and 3 d Captivator the most responding flies (*χ*^2^ = 27.04, df = 1, *P *< 0.0001: Fig 5B). The two most attractive aged Captivator baits (3 and 4 d) did not discriminately attract female or male *M. domesti*ca. Higher numbers of *M. domestica* females were attracted to 1, 2, and 7 d Captivator (Table 1). Only 0 d Captivator attracted a significantly higher number of male *M. domestica* in comparison to females (Table 1).

**Table 1.** Mean number of male and female flies responding to Captivator fly baits aged 0, 1, 2, 3, 4 and 7 days. Flowrates used were 0.08 m^3^/min for *C. macellaria* and *S. bullata* trials and 0.19 m^3^/min for *M. domestica* experiments. Statistical analyses were performed using a generalized linear model regression with Poisson distribution (GLM). The two factors evaluated were treatment and sex, with counts from 8 distinct biological replicates resulting in a consistent calculated degree of freedom (df) of 29 for each aged bait tested.

Species	Sources	GLM results of female v. male capture rates		
		Mean no. flies captured (± SEM)	*z*	*P>|z|*
***Cochliomyia macellaria***	0 d Captivator	Males: 0 (±0.16) Females: 1 (±0.50)	−1.349	0.18
	1 d Captivator	Males: 0 (±0.16) Females: 1 (±0.31)	−1.549	0.12
	2 d Captivator	Males: 0 (±0.18) Females: 3 (±0.80)	−3.677	<0.001
	3 d Captivator	Males: 1 (±0.41) Females: 2.38 (±0.53)	−3.225	0.001
	4 d Captivator	Males: 1 (±0.35) Females: 2 (±0.61)	−2.464	0.02
	7 d Captivator	Males: 0 (±0.17) Females: 2 (±0.19)	−3.022	0.003
***Musca domestica***	0 d Captivator	Males: 4 (±0.53) Females: 1 (±0.46)	5.297	<0.001
	1 d Captivator	Males: 3 (±1.01) Females: 7 (±0.38)	−2.953	0.003
	2 d Captivator	Males: 1 (±0.60) Females: 7 (±0.90)	−4.639	<0.001
	3 d Captivator	Males: 7 (±1.24) Females: 9 (±0.75)	−1.624	0.10
	4 d Captivator	Males: 8 (±1.09) Females: 9 (±1.18)	−0.616	0.54
	7 d Captivator	Males: 2 (±0.58) Females: 6 (±1.28)	−3.851	<0.001
***Sarcophaga bullata***	0 d Captivator	Males1 (±0.18) Females: 3 (±1.61)	−3.470	0.001
	1 d Captivator	Males: 2 (±0.53) Females: 2 (±0.46)	0.392	0.70
	2 d Captivator	Males: 2 (±0.53) Females: 4 (±0.63)	−2.571	0.01
	3 d Captivator	Males: 0 (±0.60) Females: 3 (±0.71)	−3.396	0.001
	4 d Captivator	Males: 1 (±0.22) Females: 3 (±0.35)	−2.738	0.01
	7 d Captivator	Males: 2 (±0.88) Females: 1 (±1.10)	1.166	0.24

**Fig. 6.**
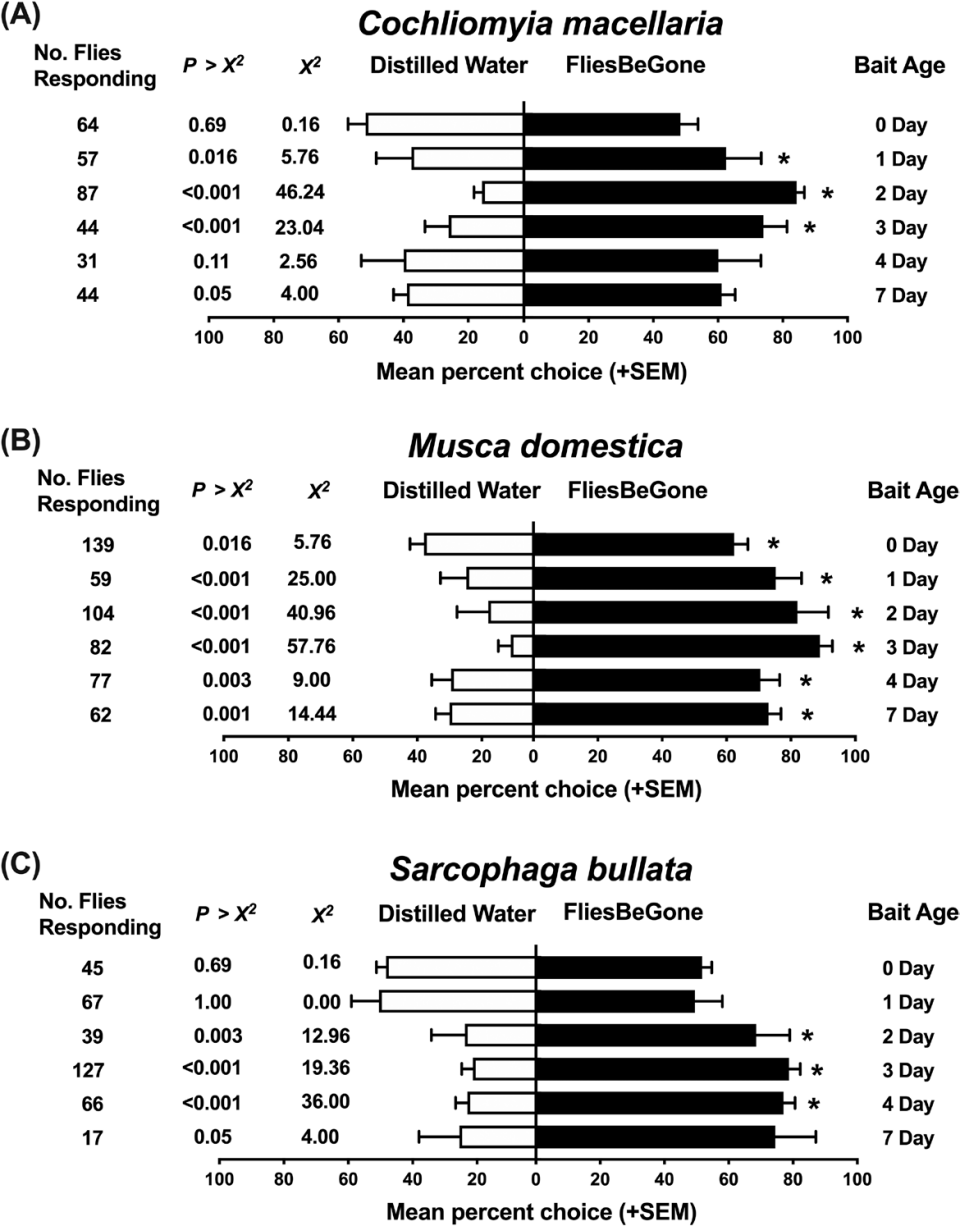
A) Mean response (+SEM) of *Cochliomyia macellaria*, B) *Musca domestica*, and C) *Sarcophaga bullata* flies in laboratory olfactometer bioassays testing 6 distinct ages of the commercial fly bait FliesBeGone, prepared according to the manufacturer’s protocol, with a distilled water control. Flowrates used were 0.08 m^3^/min for *C. macellaria* and *S. bullata* trials and 0.19 m^3^/min for *M. domestica* experiments. The release rate of FliesBeGone was 1.5 ± 0.3 mg/min for all ages tested. The observed number of responding flies (no. of flies responding) represents the combined count of eight distinct biological replicated for each distilled water treatment. Statistical analyses were performed using an adjusted *χ*^2^ analysis (*P < 0.05).

*C. macellaria*, *M. domestica*, and *S. bullata* all exhibited significant preference for 2 and 3 d FliesBeGone in comparison to distilled water in olfactometer bioassays (Fig. 6). Additionally, *C. macellaria* were significantly attracted to 1 d FliesBeGone (Fig. 6). *M. domestica* were significantly attracted to all ages of FliesBeGone, and *S. bullata* significantly attracted to 4 d FliesBeGone in comparison to distilled water. The sexual dichotomy of filth fly attraction to variant ages of FliesBeGone was unique to each species tested. *C. macellaria* presented with the most discrepancies between female and male responses. Every age of FliesBeGone tested was significantly more attractive to female *C. macellaria* in comparison to males except for 7 d FliesBeGone (Table 2). There was no significant difference between female and male responses for 7 d FliesBeGone. In contrast, only 2 d FliesBeGone attracted a higher number of female *M. domestica*, and 3 d FliesBeGone a higher number of female *S. bullata* (Table 2).

**Table 2.** Mean number of male and female flies responding to FliesBeGone fly baits aged 0, 1, 2, 3, 4, and 7 days. Flowrates used were 0.08 m^3^/min for *C. macellaria* and *S. bullata* trials and 0.19 m^3^/min for *M. domestica* experiments. Statistical analyses were performed using a generalized linear model regression with Poisson distribution. The two factors evaluated were treatment and sex, with counts from 8 distinct biological replicates resulting in a consistent calculated degree of freedom (df) of 29 for each aged bait tested.

Species	Sources	GLM results of female v. male capture rates		
		Mean no. flies captured (± SEM)	*z*	*P>|z|*
***Cochliomyia macellaria***	0 d FliesBeGone	Males: 1 (±0.25) Females: 3(±0.48)	−3.139	0.002
	1 d FliesBeGone	Males: 1(±0.42) Females: 3 (±0.76)	−2.510	0.012
	2 d FliesBeGone	Males: 3 (±0.76) Females: 8 (±0.67)	-4.463	<0.001
	3 d FliesBeGone	Males: 1(±0.32) Females: 3 (±0.60)	−3.464	0.001
	4 d FliesBeGone	Males: 0 (±0.16) Females: 2 (±0.46)	2.519	0.01
	7 d FliesBeGone	Males: 1 (±0.54) Females: 2 (±0.79)	-1.908	0.56
***Musca domestica***	0 d FliesBeGone	Males: 5 (±0.67) Females: 6 (±0.93)	−0.654	0.51
	1 d FliesBeGone	Males: 2 (±0.48) Females: 3 (±0.50)	−1.428	0.15
	2 d FliesBeGone	Males: 3 (±1.00) Females: 8 (±2.14)	−3.948	<0.001
	3 d FliesBeGone	Males: 4 (±0.48) Females: 5 (±0.63)	-1.408	0.16
	4 d FliesBeGone	Males: 8 (±1.08) Females: 9 (±1.18)	−2.467	0.02
	7 d FliesBeGone	Males: 2 (±0.46) Females: 4 (±1.13)	−2.031	0.04
***Sarcophaga bullata***	0 d FliesBeGone	Males: 1 (±0.23) Females: 2 (±0.16)	−1.034	0.30
	1 d FliesBeGone	Males: 1 (±0.46) Females: 3 (±0.71)	−2.143	0.032
	2 d FliesBeGone	Males: 3 (±0.85) Females: 3 (±0.82)	−0.576	0.57
	3 d FliesBeGone	Males: 2 (±0.45) Females: 11 (±2.00)	−6.436	<0.001
	4 d FliesBeGone	Males: 2 (±0.48) Females: 5 (±1.40)	−3.194	0.001
	7 d FliesBeGone	Males: 1 (±0.42) Females: 1 (±0.23)	−0.575	0.57

## Discussion

Olfactometers are valuable tools to investigate responses of insects to attractants, repellents, and a variety of odorants (Katsoyannos et al. 1980). One advantage of spatial olfactometers is that the design allows insects to take flight, which does require sufficient space. The dual choice cube design developed by Flint and Tomberlin (2021) and the I-box wind tunnel (Moophayak et al. 2013) both allow flight. For our study, small modifications to the olfactometer design resulted in a robust system that increased the response rate of filth flies and generated discernable choice data and allowed for increased replication. Four olfactometers were in operation simultaneously, each containing 30 to 50 adult flies, depending on the availability of test subjects. Fresh air passed over the attractant or the water control in either collection chamber A or B and into the olfactometer holding the flies (Figs. 1 and 2). Flies responded to the stimulant by flying upward and into the preferred collection chamber, allowing for easy quantification of responses. Air was removed from the olfactometer through vacuum ports in the base. Using this system, we were able to replicate behavioral assays efficiently, allowing well-powered studies to evaluate responses of *C. macellaria*, *M. domestica*, and *S. bullata*.

The behavioral response of filth flies to Captivator and FliesBeGone baits paired against distilled water was unique to each species tested. When paired with water, *C. macellaria* and *S. bullata* were significantly more attracted to FliesBeGone, whereas *M. domestica* preferred Captivator. Both of these baits contain odors of putrid nature or contents related to decaying organic matter. It is clear that these species-specific responses to Captivator and FliesBeGone are due to differences in bait composition and subsequent VOC profiles. While the FliesBeGone bait components are proprietary information, the SDS label lists eggs as an ingredient, which would contribute as a fly attractant. https://fliesbegone.com/pages/sds-information. The Captivator active ingredients are listed on the label. They contain eggs as well, which is a known filth fly attractant. This along with additional odors that are considered attractive to *M. domestica* (Z-9-tricosene, indole, trimethylamine). This could explain why *M. domestica* preferred Captivator (https://www.starbarproducts.com/all-products/traps/captivator-fly-trap). Otherwise, it is unclear which differences between these two commercial fly baits are causing the observed species-specific responses.

In general, aging the commercial baits did not increase attraction. It is important to note that while the microorganisms are important generators of common decomposition VOCs, there is no one microorganism or VOC dictating filth fly behavioral responses but a profile of odors that gets the better fly response (Chaudhury et al. 2010, Hung et al. 2015, 2020). The observed decrease in filth fly attraction to 7 d Captivator and FliesBeGone attractants may be explained by the baits becoming increasingly concentrated (putrefaction and evaporation) and no longer representative of a fertile nutrient source or oviposition site.

The sexual dichotomy of filth fly responses to Captivator and FliesBeGone may be explained by the distinct behaviors of males and females as males engaging in mating behavior and females’ oviposition behavior. It is plausible that the VOC profiles of Captivator and FliesBeGone contain more oviposition cues, and thus attract a predominately higher number of female filth flies. The consistent male *M. domestica* behavioral response to Captivator and FliesBeGone may be a result of their aggressive reproductive behavior, actively chasing and colliding with conspecifics in an attempt to copulate. This behavioral phenomenon may be explained by higher levels of Z-9 tricosene (muscalure) known to increase in flies held in colony, as was ours, for several generations (Adler et al. 1984, Noorman and Den Otter 2001, Darbro et al. 2005).

In summary, our spatial olfactometer is a valuable tool to measure fly responses to attractants. We provided sufficient space within the olfactometer to allow for a more natural flight response, and the replication with 8 chambers means 2 products can be tested at a given time and replicated 4 times. This also allows control over day-to-day, or even within a day, sources of unexplained variation. Future studies will allow releases of two or more species within olfactometers or measure responses to conditioned or modified insects. We can also conclude that flies respond best when air flowrates are lower, that commercial Captivator and FliesBeGone attractants effectively attract *M. domestica* and that *C. macellaria* and *S. bullata* are slightly more attracted to FliesBeGone baits. We also showed that aging of these commercial baits do not appear to be advantageous.

## Supplementary Material

ieaf020_suppl_Supplementary_Tables_S1
