# Supplementary material for: Response of adult Cochliomyia macellaria, Musca domestica, and Sarcophaga bullata (Diptera: Calliphoridae, Muscidae, Sarcophagidae) to odors produced by commercial fly baits in a two-choice olfactometer bioassay
Source: J Insect Sci. 2025 Apr 8;25(2):11. doi: 10.1093/jisesa/ieaf020 (PMC11977110; doi:10.1093/jisesa/ieaf020)
Supplement: ieaf020_suppl_Supplementary_Tables_S1 [file ieaf020_suppl_supplementary_tables_s1.docx]

**Supplemental Table 1.** Poisson distribution of experimental variance for responses of *Cochliomyia macellaria*, *Musca domestica* and *Sarcophaga bullata* to commercial baits in laboratory olfactometer bioassays and standardization experiments.

| **Species** | **Source** | **DF** | ***χ^2^*** | ***Pr* > *χ^2^*** |
| --- | --- | --- | --- | --- |
| *Cochliomyia* | Captivator |  |  |  |
| *macellaria* | Treatment | 6 | 35.940 | <0.001 |
|  | Position | 1 | 0.044 | 0.83 |
|  | Cage | 3 | 3.966 | 0.27 |
|  | Day | 1 | 0.183 | 0.67 |
|  | FliesBeGone - Flowrate |  |  |  |
|  | Flowrate | 1 | 15.906 | <0.001 |
|  | Treatment | 1 | 10.332 | 0.001 |
|  | Position | 1 | 1.418 | 0.23 |
|  | Cage | 3 | 5.052 | 0.17 |
|  | Day | 1 | 1.032 | 0.31 |
|  | FlieBeGone |  |  |  |
|  | Treatment | 6 | 110.785 | <0.001 |
|  | Position | 1 | 0.022 | 0.88 |
|  | Cage | 3 | 3.599 | 0.31 |
|  | Day | 1 | 1.132 | 0.29 |
| *Musca* | Captivator - Flowrate |  |  |  |
| *domestica* | Flowrate | 4 | 103.465 | <0.001 |
|  | Treatment | 1 | 141.633 | <0.001 |
|  | Position | 1 | 0.028 | 0.87 |
|  | Cage | 3 | 0.060 | 0.81 |
| ­ | Day | 1 | 3.650 | 0.06 |
|  | Captivator |  |  |  |
|  | Treatment | 6 | 267.128 | <0.001 |
|  | Position | 1 | 3.361 | 0.07 |
|  | Cage | 3 | 6.471 | 0.09 |
|  | Day | 1 | 0.099 | 0.75 |
|  | FliesBeGone |  |  |  |
|  | Treatment | 6 | 110.433 | <0.001 |
|  | Position | 1 | 2.654 | 0.10 |
|  | Cage | 3 | 0.467 | 0.93 |
|  | Day | 1 | 1.109 | 0.29 |
| *Sarcophaga* | FliesBeGone - Flowrate |  |  |  |
| *bullata* | Flowrate | 3 | 84.129 | <0.001 |
|  | Treatment | 1 | 66.091 | <0.001 |
|  | Position | 1 | 0.162 | 0.69 |
|  | Cage | 1 | 3.966 | 0.50 |
|  | Day | 4 | 6.522 | 0.16 |
|  | FliesBeGone |  |  |  |
|  | Treatment  Position  Cage  Day | 6  1  3  3 | 149.019  1.244  0.884  6.770 | <0.001  0.27  0.83  0.08 |
|  | Captivator  Treatment  Position  Cage  Day | 6  1  1  1 | 24.996  0.971  0.014  0.637 | <0.001  0.32  0.32  0.43 |
